# Overcoming Political Fragmentation: The Potential of Meso-Level Mechanisms: Comment on "Integration or Fragmentation of Health Care? Examining Policies and Politics in a Belgian Case Study"

**DOI:** 10.34172/ijhpm.2022.7075

**Published:** 2022-03-12

**Authors:** Carolyn Steele Gray

**Affiliations:** ^1^Bridgepoint Collaboratory for Research and Innovation, Lunenfeld-Tanenbaum Research Institute, Sinai Health, Toronto, ON, Canada.; ^2^Institute of Health Policy, Management and Evaluation, Dalla Lana School of Public Health, University of Toronto, Toronto, ON, Canada.

**Keywords:** Health Policy, Integrated Care, Change Mechanisms, Belgium

## Abstract

Martens and colleagues’ paper "Integration or Fragmentation of Health Care? Examining Policies and Politics in a Belgian Case Study," offers an in-depth examination of integrated care policy efforts in Belgium. A key finding in this case study was that political fragmentation proved too great an obstacle for integration efforts. In this commentary, I draw on the organizational behaviour and integrated care literatures to suggest how meso-level mechanisms related to sensemaking, distributive leadership, and evaluation could help overcome policy (or macro) level challenges like those experienced in Belgium. The commentary also suggests we need to consider and address both the process and normative challenges in these transformation efforts.

 Increasingly, health systems globally have been working towards more integrated health and social care service delivery. Martens and colleagues’^1^ case study of Belgium’s policy transformation over the last decade to establish integrated care offers an in-depth view of this process under a federated government. This case study explores three major policy shifts that were put in place at federal and regional levels to advance integrated care delivery. While some progress was made, mostly in the form of pilot projects, the analysis in this study uncovers a myriad of challenges related to structures and power imbalances that ultimately thwarted significant transformation. Importantly, this paper highlights tensions between the nature of a federal structure and the aims of integrated care, concluding that, for Belgium, “political fragmentation trumps care integration.”

 Martens and colleagues’^1^ policy analysis uncovers several key challenges. First, there were disagreements regarding the roles and responsibilities of federal and federated groups, resulting in some arguing the integrated care transformation was unconstitutional. Second, there were difficulties in establishing strong leadership that could bridge the divide between political structures and work on the ground. Activities like building trust-based relationships across stakeholders, supporting an adaptive and experimental process, and ensuring that the purpose of the transformation effort was clear to all stakeholders were seen to be missing in the Belgium example. Finally, evaluation and performance frameworks created an overly controlling environment, but were also insufficient to produce data that could effectively demonstrate impact. These three challenges point to an inherent problem facing jurisdictions who seek to adopt integrated care more widely; namely, that there is a disconnect between the core aims of integrated care which strives for greater connection within an environment that is mired in division.

 These types of political tensions have been identified in other studies of integrated care.^2,3^ In their study of 17 integrated care case studies from 8 European countries, Looman and colleagues identified similar tensions when seeking to implement integrated care.^4^ This large comparative case study reveals 10 mechanisms that drive implementation, chief among which was “engaging in alignment work” which helped to overcome the challenge that “macro-level policies are often not supportive of integrating care” (p. 8). In these examples alignment work occurred largely at the meso level, namely through the work of leaders and managers who sought to bridge tensions, work around challenges, and foster collaboration, relationships, and trust. The remainder of this commentary explores how meso-level mechanisms could potentially overcome the challenges related to political fragmentation found in Martens and colleagues’^1^ study.

## Clarifying Objectives

 One of the challenges experienced in the Belgian transformation example was around misunderstandings between different groups working to advance integrated care. Among the issues noted was a lack of clarity about where reform efforts were supposed to lead, and how these efforts aligned to existing funding and governance models. A complex adaptive systems approach helps us to understand how having a clear understanding and purpose amongst stakeholders can be central to transformation efforts.

 Complex adaptive systems theory encourages us to see these transformations as dynamic processes that are shaped by interactions and relationships.^5,6^ Essential to this process is how meaning is shared through interactions across networks to help ensure diverse stakeholders are pulling in the same direction. Consequently, complex adaptive systems theory has demonstrated the foundational role of *sense-making* in transformation efforts^7^ which can provide clarity on the purpose and intention of changes across diverse stakeholder groups. In their study of healthcare reforms in Quebec, Denis et al^8^ identify an important tension between sense-making and organizing. To manage the identified tension, organizations undergoing major transformations in Quebec assigned particular managers as *sense-makers-in-chief *who could help understand and shape the strategic change on the ground, while staying connected to the wider vision of the transformation. Ultimately, the role of the *sense-makers-in-chief* was viewed as a cooperative role, where they collectively construct and circulate new meanings within and across their organizations. These *sense-makers-in-chief* helped to ensure diverse stakeholders within the network had a shared understanding of the purpose and intent of the changes ahead of them. Having consistent communication between federal bodies and those leading transformation efforts, possibly through establishing positions whose job is specifically to ensure shared understanding like a *sense-maker-in-chief*, could help to improve clarity of purpose which was an important gap for Belgium.

## Models of Distributed Leadership

 Leadership gaps were a critical challenge facing political reform efforts in Belgium. Two of the 10 mechanisms for implementing integrated care in the Looman et al comparative case study are focused on the role of leadership, with a number of the other mechanisms relying on the work of leaders. Leadership in networks has been noted as being more challenging than leadership in single organizations, and requires a shift towards a distributed leadership model, where responsibility is shared.^4^ Sharing leadership across the network could address challenges of leadership turnover experienced in the Belgian case, as well as address issues related to power imbalances across stakeholders. A distributed leadership approach is aligned with a model of New Power^9^ which is relational, collaborative, and fluid as compared to a model of Old Power which is transactional and hierarchical.^10^ In a New Power approach there is an alignment to common goals, helping to ensure multiple players needed to enable change are on board and pulling in the same direction^11^; addressing some of the key challenges of misalignment that occurred in Belgium.

## Evaluating and Measuring With Purpose

 Performance reporting requirements and evaluation challenges marked a significant barrier when implementing new initiatives, and to scaling and spreading promising pilots in Belgium. Looman’s mechanisms around information and research speak to the need to create continuous feedback loops and monitoring of progress.^4^ Not to constrain activities as was the case in Belgium, but to enable adaptability and incremental change. Leveraging real-time performance data to inform iterative improvement towards value-based healthcare is a cornerstone of a Learning Health System approach. In these models,’ evaluation is sought through routinized learning cycles that bring together practice, decision, and knowledge related data to continuously improve processes.^12^ In the World Health Organization (WHO) flagship report on Learning Health Systems, it is argued that integration of evaluation data into health system operations can support ongoing institutional learning around individual actions, program theory or assumptions, and system structures.^13^ The important distinction here is the underlying reason for the measurement of performance – not to constrain, but to grow. This requires “a holistic appraisal of value” which is jointly determined by partners regarding how well community needs are being met. Meso level mechanisms like strong leadership, partnership, and organizational priority setting have been identified as important enablers for this approach to evaluation that is more aligned with a learning health system approach.^14^

## Addressing Both Process and Normative Challenges

 Looking across these meso-level mechanisms, one can argue they pull on two important levers of change: process and norms. Focusing on the structures and actions of systems and individuals alone can miss catching the norms, values, and beliefs that hold-up and entrench the status quo. The act of sense-making not only can clarify the aims and purpose of a transformation, but also helps to understand what is important and meaningful to those individuals engaging in the work and uncover value-systems that have driven political decisions in the past. Distributed leadership requires both a procedural change in who makes decisions-when, but also a belief that we need to shift the locus of power in our systems to one that is collaborative and shared. And a Learning Health System mentality both builds a process for feedback and believes in the importance of continuous learning, so that performance measurement is not a means of control, but an avenue for improvement.

 At the end of Martens and colleagues’ paper they identify that they did not attend to a couple of factors that may also influence the trajectory of integrated care implementation. Two noted in this paper include goal-oriented care and digital health technologies. These two factors may be particularly important as they can also pull process and norm levers of change, and both may be powerful tools to support implementation of integrated care. First, with regard to goal-oriented care, patient-goals can act as a catalyst for micro- meso-, and macro-level integration through enabling both functional and normative processes.^15^ Second, digital health technologies can similarly catalyze both process and normative change within health and social care systems and can additionally help attend to the evaluation and performance challenge identified in the Belgian example.^16^ While the mechanisms discussed above operate largely at the meso level, goal-oriented care and digital health can activate change at all levels, potentially accelerating transformation.

 In addition to these specific examples, other solutions to process and normative challenges may emerge through the natural process of transformation in a complex system. Returning to complex adaptive systems theory, the process of self-organization, in which individuals fall into naturally occurring patterns through their interactions, can help to address shared needs in unpredictable ways.^17^ In Lanham and colleagues’ comparative case study of health system transformation, it was found that self-organization played a central role in the scale-up and spread of interventions, through iterative adjustments in response to unexpected challenges.^17^ From this view, a key meso-level mechanisms occurs at the level of individual and organizational interaction, in which networks can come together to address unpredictability and uncertainty through adoption of new, maybe unexpected, processes and actions.

 To conclude, this commentary does not suggest that any one of the approaches offered here will constitute the silver-bullet solution to the important political fragmentation problem identified by Martens et al. Those types of entrenched political structures are firmly in place and will be hard to dismantle. Instead, the above mechanisms can be viewed as tools to help coax more pliability out of those structures; bending the girders when possible or building around them when needed.

## Ethical issues

 Not applicable.

## Competing interests

 Author declares that she has no competing interests.

## Author’s contribution

 CSG is the single author of the paper.
